# Editorial: Regulation of synaptic structure and function

**DOI:** 10.3389/fnmol.2022.1060367

**Published:** 2022-10-14

**Authors:** Zhiyong Shao, Yang Yang, Zhitao Hu

**Affiliations:** ^1^State Key Laboratory of Medical Neurobiology and MOE Frontiers Center for Brain Science, Institutes of Brain Science, Fudan University, Shanghai, China; ^2^School of Life Science and Technology, ShanghaiTech University, Shanghai, China; ^3^Queensland Brain Institute, Clem Jones Centre for Ageing Dementia Research (CJCADR), The University of Queensland, Brisbane, QLD, Australia

**Keywords:** synapse, synaptic formation, synaptic plasticity, synaptic function, *C. elegans*, Wnt, SNARE, synaptic transmission

## Introduction

Synaptic structure and function are fundamental for normal function of the nervous system. Synapses contain pre-synaptic terminals, synaptic cleft, and post-synaptic structures. Upon stimulation, synaptic vesicles in the pre-synaptic terminals fuse with the plasma membrane and release neurotransmitters to the synaptic cleft. The neurotransmitters then bind to their receptors on the post-synaptic membrane, leading to excitation or inhibition of the post-synaptic neurons, determined by the types of neurotransmitters and receptors. These brain functions require precise regulation of synaptic connections. In this Research Topic, we collected 19 research and review papers to address how synaptic structure and functions are regulated. Here we summarize these papers to guide readers through this Research Topic.

## Regulation of synaptic structure

During the process of synaptic development, neurons first form complex neurites including axons and dendrites (Keyser, 1983; Gallo, 2013; Robichaux and Cowan, 2014). Yang et al. discovered that a novel adaptor for the RING-domain type ubiquitin E3 ligase, CG5003, is required for the axon development in the *Drosophila* Mushroom Body, providing further evidence that protein homeostasis is critical for axonal and synaptic development. In addition to ubiquitination, synaptic proteins are subjected to many other post-translational modifications such as small ubiquitin-like modification (SUMO). SUMOylation has been shown to modify and regulate synaptic proteins (Loriol et al., 2012; Daniel et al., 2017; Henley et al., 2021). To better understand the role of SUMOylation in synaptic sites, Pronot et al. performed SUMOylation proteomic analysis with synaptic fraction in rat brains and found that about 18% of synaptic proteins are SUMOylated, indicating SUMOylation in synaptic structure is very common.

Synaptic formation is regulated by secreted and cell adhesion molecules, such as Wnt, neurexin and Ephrin-Eph receptors (Salinas, 1999; Contractor et al., 2002; Lai and Ip, 2009; Poon et al., 2013; Sudhof, 2018). The role of Wnt signaling in synaptic development has been shown to be highly conserved (Salinas, 1999; Beretta et al., 2011; Shi et al., 2018). Teo and Salinas reviewed the recently advance of Wnt function in excitatory synaptogenesis, with focus on the role of Wnt5a and Wnt7a. Additionally, they summarized the role of neural activity in synaptic formation through Wnt7a/b. In mammals, through alternative splicing, neurexin genes produce diverse isoforms in different neuron types (Treutlein et al., 2014; Sudhof, 2017; Lukacsovich et al., 2019; Gomez et al., 2021). Previous studies indicated the alternative splicing of neurexin is activity-dependent (Gorecki et al., 1999; Rozic-Kotliroff and Zisapel, 2007; Iijima et al., 2011; Ding et al., 2017). Liakath-Ali and Südhof found that in cortical cultures and *in vivo* experiments, the previous finding about activity induced Nrxn1-SS4 specific splicing is most likely a secondary effect of activity-induced cell death. This study suggests that the link between neuronal activity and Nrxn1-SS4 splicing requires more sophisticated data. Li et al. found that EphB2-mediated dendritic spinogenesis is regulated by a scaffold protein, ligand of Numb protein X 1, which functions through maintaining EphB membrane localization. These studies further reiterate that secreted and cell adhesion molecules play vital roles in synaptic formation.

Recent studies indicate that non-coding RNA plays various roles in nervous system, including in synaptic formation. Wakatsuki and Araki summarized their recent finding about a special non-coding vault RNAs regulating synaptic formation through upregulating MEK1-ERK signaling pathway (Wakatsuki et al., 2021), which provides a novel synaptogenesis signaling pathway.

In addition to genetic factors, synaptic connections are also regulated by environmental conditions including ion concentration or drug usage. Zinc homeostasis has been shown to associate with neurologic disorders. Mo et al. found that in cultured neurons, high concentration of zinc results in synaptic formation and function defects through reducing the expression of PTPRM. It would be interesting to ask how PTPRM regulates synaptic formation. Drugs such as antibiotics or psychedelics could potentially affect synaptic formation and plasticity. Perna et al. found that perinatal penicillin exposure results in excessive synaptic pruning and lower dendritic spine density in the cortical region in mice, suggesting that the usage of antibiotics in early-life has an important effect on brain development and function. Consistently, Lukasiewics et al. summarized the critical role of exposure to another drug, psychedelics, in synaptic formation and neuronal plasticity. These studies provide insights into the mechanisms underlying the effects of drugs on the brain and will guide future drug development and usage.

Sexual dimorphism is observed at different levels of nervous system, which could explain the sex difference of neurological disorders. Uhl et al. reviewed the sexual dimorphism, with a special focus on synaptic density, morphology and molecular composition. The review summarized an advanced understanding of sexual synaptic dimorphism and potential underlying regulatory mechanisms. Taking advantage of the simplicity of the nematode *C. elegans*, Yan et al. analyzed the sexual synaptic structure and function dimorphism *in vivo* and revealed that at the neuromuscular junction (NMJ), the cholinergic synaptic density, the frequency of spontaneous neurotransmitter release, and the locomotory velocity of males are higher than those of hermaphrodites. Those studies provide cellular and molecular evidence underlying physiological and pathological sexual difference.

Synaptic maintenance and regeneration are essential for neural function. Huang et al. reviewed the mechanisms underlying NMJ degeneration and regeneration after denervation and the potential therapeutic strategies, which summarized recent advances in the maintenance and repair of NMJ synaptic connection.

## Regulation of synaptic function

One of the most important functions of synaptic connection is to relay information from pre-synaptic to post-synaptic neurons. For example, in the sensory circuitry, sensory neurons are activated upon stimulation, which triggers the pre-synaptic synaptic vesicle fusion with the plasma membrane and the release of neurotransmitters and neuromodulators, through which the information from sensory neurons is passed onto their downstream targets.

*C. elegans* has served as an excellent model in studying neuronal activity and functional circuits (Bargmann et al., 1990; Kaplan, 1996; Bargmann, 2006; Dixit and Bhattacharya, 2021; Ferkey et al., 2021). To understand how sensory neurons respond differently to different odor concentrations, Cheng et al. analyzed four olfactory neurons AWA, AWB, AWC, and ASH. They found that while AWC shows no difference in response to different concentrations of isoamyl alcohol (IAA), the other three olfactory neurons display concentration-dependent responses, which uncovers the mechanisms underlying animal sensing different odor concentrations. Olfactory sensory neurons can adapt long-lasting or repetitive stimulation. Chen et al. found that adaptation to odor stimuli requires L-type voltage-gated calcium channel (L-VGCC) EGL-19 in Amsh glia in *C. elegans*, which highlights the important role of VGCC and glia in modulating sensory transduction. Ion channels are essential for depolarizing cell membrane and triggering behaviors. Yu et al. showed that A-type motoneurons display intrinsic rhythmic activity, which requires Na^+^ leak channels, VGCC and voltage-gated K^+^ channel (Kv4). Bhat et al. reviewed the important roles of neuropeptides in regulating neural activity and various behaviors. Those studies demonstrate that neuronal activity and therefore behaviors are regulated by external and internal factors.

In muscles, upon plasma membrane depolarization, L-VGCC undergoes conformation change and triggers calcium release by opening ryanodine receptors (RyR) and calcium channels on endoplasmic reticulum (ER). Muscle cell plasma membrane invaginates deep into the cell forming junctional membrane complex (JMC) with ER, which is critical for depolarization-induced calcium release. Piggott and Jin reviewed the key findings from both invertebrates and vertebrates about the role of the highly conserved junctophilins in JMC formation and function in muscles and the afterhyperpolarization (AHP) in neurons, which provided a comprehensive understanding of the function of junctophilins and JMC.

Calcium influx in pre-synaptic terminals promotes synaptic vesicle priming and fusion with the plasma membrane mediated by the SNARE complex (Bargmann, 1993; DiAntonio et al., 1993; Sollner et al., 1993; Littleton and Bellen, 1995; Sudhof and Rothman, 2009). During the process of SNARE complex, the Munc18-1/syntaxin-1 complex is disassembled. Gong et al. showed that opening syntaxin-1 linker is critical to initiate SNARE complex assembly, and the extension of Munc18-1 domain 3a regulated by Munc13 is required for synaptobrevin-2 and syntaxin-1 interaction and SNARE complex assembly. In addition to Munc18 and Munc13, there are a large cohort of SNARE regulatory proteins (SRPs) regulating SNARE assembly at the right time and right place. Sauvola and Littleton summarized the current models about how SRPs regulate SNARE assembly in the pre-synaptic sites to trigger neurotransmitter releases, particularly emphasizing the genetic findings from invertebrates such as *C. elegans* and *Drosophila*. The review provided a detail picture of regulation of pre-synaptic vesicle release.

## Conclusion remarks

In summary, this Research Topic collected a series of research and review articles that are related to synaptic structure and function regulation, and advanced our knowledge on synapses and neural circuitry.

## Author contributions

ZS drafted the manuscript. ZS, YY, and ZH edited the manuscript, contributed to the Research Topic, and approved the publication of this Editorial. All authors contributed to the article and approved the submitted version.

## Funding

This work was supported by the Ministry of Science and Technology, People's Republic of China (2021YFA0909303 to ZS), National Natural Science Foundation of China (31872762 and 32170828 to ZS and 31970960 to YY), the Shanghai Municipal Science and Technology Major Project (no. 2018SHZDZX01) and ZJLab (to ZS), and National Health and Medical Research Council Project grant (GNT1122351 to ZH).

## Conflict of interest

The authors declare that the research was conducted in the absence of any commercial or financial relationships that could be construed as a potential conflict of interest.

## Publisher's note

All claims expressed in this article are solely those of the authors and do not necessarily represent those of their affiliated organizations, or those of the publisher, the editors and the reviewers. Any product that may be evaluated in this article, or claim that may be made by its manufacturer, is not guaranteed or endorsed by the publisher.
